# Impact of Human Papillomavirus Primary Cervical Screening on Referral Rates and Outcomes for People Referred to Colposcopy for Clinical Reasons

**DOI:** 10.1111/ajo.70184

**Published:** 2026-10-04

**Authors:** Adibah Khan, Katyana Gullery, Georgina Barber, Judy Ormandy

**Affiliations:** ^1^ Department of Obstetrics and Gynaecology University of Otago Wellington, Te Whatu Ora – Capital, Coast and Hutt Valley Wellington New Zealand

**Keywords:** cervical intraepithelial neoplasia, colposcopy, human papillomavirus DNA tests, mass screening, postcoital bleeding

## Abstract

Colposcopy is most commonly indicated for people with screen‐positive cervical screening tests. However, clinical reasons such as post‐coital bleeding (PCB) or an abnormal appearing cervix can also prompt referral. Aotearoa New Zealand (AoNZ) transitioned from a cytology‐based to an HPV‐based cervical screening programme in September 2023.

**Aim:**

To assess the incidence of high‐grade or malignant pathology in people seen in Capital & Coast colposcopy clinic for clinical reasons and to evaluate the impact of HPV‐based primary screening on these referral patterns and outcomes.

**Methods:**

A retrospective review was undertaken of individuals referred to Capital & Coast colposcopy services for clinical indications. Two cohorts were analysed: Cohort 1 (June 2021–June 2023), during cytology‐based screening and Cohort 2 (November 2023–November 2025), following implementation of HPV‐based screening. Clinical outcomes were obtained from hospital databases.

**Results:**

Referrals for clinical reasons decreased significantly following the introduction of HPV screening, from 494/1699 (29%) in Cohort 1 to 252/2809 (9%) in Cohort 2 (*p* < 0.01). High‐grade or malignant pathology was detected less frequently in Cohort 2 (0/252 (0%) vs. 7/494 (1.4%)), although this was not statistically significant (*p* = 0.10).

**Conclusion:**

Referrals to colposcopy for clinical reasons decreased substantially following the introduction of HPV‐based primary screening, with no high‐grade or malignant pathology detected among those referred in Cohort 2 who did not meet screening‐based referral criteria. While reassuring, HPV testing does not detect all cervical disease. Clinical assessment remains important for persistent or concerning symptoms or abnormal cervical findings, including consideration of HPV‐independent disease.

## 1 Introduction

Colposcopy is an essential component of cervical screening programmes to detect cervical precancer and cancer. Its main objective is the detection of high‐grade disease, defined as cervical intraepithelial neoplasia grade 2+ (CIN2+) or high‐grade squamous intraepithelial lesions (HSIL). Most colposcopy referrals arise from screen‐positive cervical screening test results [1]; however, clinical symptoms such as post‐coital bleeding (PCB), intermenstrual bleeding (IMB) or an abnormal‐appearing cervix remain important indications because they may signal premalignant or malignant disease [2]. Although most people presenting with abnormal vaginal bleeding do not have pre‐malignant or malignant disease, PCB, IMB and other irregular bleeding patterns can be associated with genital tract malignancy or premalignant changes, as well as a wide range of benign causes including polyps, adenomyosis, leiomyomas, coagulopathies, ovulatory disorders, endometrial conditions and iatrogenic factors [2]. PCB is a recognised early symptom of cervical cancer [2]. While the absolute risk of cancer in patients with PCB is low, a small but clinically meaningful proportion have CIN2/3 or invasive disease, reinforcing the importance of timely colposcopic assessment [3].

Prior to September 2023, Aotearoa New Zealand (AoNZ) had a cytology‐based cervical screening programme with some reflex HPV testing [2]. In September 2023, AoNZ transitioned to HPV primary screening with reflex cytology, alongside the introduction of self‐collected vaginal swabs [2]. HPV testing provides greater sensitivity than cytology for detecting high‐grade lesions, and its acceptability to participants is enhanced by the availability of self‐sampling [2, 4]. AoNZ was the first high‐income country to transition directly to HPV self‐testing as the primary screening method for its cervical cancer prevention programme [4]. Since this change, approximately 80% of cervical screening tests are now self‐collected, with 20% clinician‐collected; the improved cervical screening coverage has resulted in increased colposcopy referrals [5, 6]. People who have HPV16 or 18 detected at primary screening are referred directly for colposcopy [2]. Those with HrHPV type ‘other’ have reflex cytology performed, and if high grade, colposcopy is indicated [2]. People who are symptomatic with abnormal bleeding have a clinician‐collected co‐test (cytology and HPV) [2].

Self‐testing is only recommended for asymptomatic participants [2]. People reporting symptoms such as PCB should have clinician administered cytology and HPV testing [2]. Many people previously referred with an abnormal cervix had normal screening tests so it was anticipated that in the context of self‐collected screening without a clinical examination, these participants would not raise clinical concern or a referral [2].

Understanding the clinical outcomes of people referred for colposcopy for clinical reasons alone is essential to ensure optimal and evidence‐based use of colposcopy resources [7]. This study therefore aims to: (1) determine the incidence of high grade (CIN2+) or malignant cervical pathology among people referred to colposcopy for clinical reasons alone in Wellington and (2) evaluate the impact of the introduction of HPV primary screening on referral volumes and clinical outcomes in this group.

## 2 Materials and Methods

This study was conducted at a tertiary obstetrics and gynaecology service within Te Whatu Ora—Capital & Coast, serving a population of approximately 540,000 [8]. A retrospective cohort analysis of colposcopy assessments performed for clinical reasons was identified from the Gynaecology Plus Database. First Special Appointments (FSAs) for colposcopy from two 2‐year cohorts were identified: Cohort 1 included those who presented between July 2021 and June 2023; Cohort 2 included those seen between November 2023 and November 2025.

Of all people attending a colposcopy FSA, those referred for clinical reasons—PCB, an abnormal‐appearing cervix, IMB, or PMB—were included in each cohort. In cases where both clinical indications and an abnormal screening test, respective screening criteria took precedence. Those who had an abnormal screening test that would not on its own generate a colposcopy referral, such as HrHPV other with normal cytology, along with clinical reasons, were included. People were excluded if the screening tests alone warranted a referral, such as HPV16 detected.

For each case, collected data included demographic characteristics (age and ethnicity), referral indications, prior HPV and cytology results, appointment outcomes such as tests performed (histology, cytology, HPV), diagnoses and treatments. Information was obtained from the Gynaecology Plus and Single Clinical Portal patient information systems. Descriptive analyses compared the total number of clinical referrals, referral indications, pre‐referral HPV testing status, and detection rates of high‐grade or malignant disease between cohorts. Demographic and clinical characteristics were compared between cohorts. Age was compared using a *t*‐test. Ethnicity and pre‐FSA test types were compared using chi‐squared tests, with a Fisher's exact test used to compare referral indications.

This project was approved by the Women's Health Research and Audit Committee at Te Whatu Ora Capital, Coast and Hutt Valley. As this was a retrospective service evaluation, formal ethics committee approval was not required.

## 3 Results

In Cohort 1, there were 1699 colposcopy first specialist assessments (FSAs), of which 494 patients (29%) met the study inclusion criteria. In Cohort 2, there were 2809 FSAs, of which 252 (9%) met the study inclusion criteria. An increase in total colposcopy first specialist assessments (FSAs) was observed following the introduction of HPV primary screening.

Demographic characteristics, referral indications, and screening test results for both cohorts are presented in Table 1. No significant differences were identified in age group or ethnicity distribution between the two cohorts. Consistent with the transition from cytology‐based to HPV‐based screening, cohort 2 was significantly more likely to have undergone HPV testing prior to referral (92% vs. 2% in Cohort 1) (*p* < 0.01). Cytology testing within the year prior to FSA occurred in 93% of cohort 2 and 99% of cohort 1 (*p* < 0.01). The most common indications for colposcopy review were PCB and a cervix thought to be clinically abnormal. There were more referrals in Cohort 1 for abnormal bleeding that wasn't post‐coital and when primary care clinicians were unable to obtain cytology.

**TABLE 1:** Demographic characteristics of people referred to colposcopy clinic ‘clinical reasons only’.

	Cohort 1—July 2021 to June 2023	Cohort 2—November 2023 to November 2025
	*n* = 494	*n* = 252
Age		*p* = 0.89
15–19	7 (1%)	3 (1%)
20–29	145 (30%)	60 (24%)
30–39	123 (25%)	84 (33%)
40–49	118 (24%)	67 (27%)
50–59	71 (14%)	27 (11%)
60–69	23 (5%)	8 (3%)
70–80	7 (1%)	3 (1%)
Ethnicity		*p* = 0.36
European	300 (61%)	146 (58%)
Māori	66 (13%)	38 (15%)
Pacific	35 (7%)	12 (5%)
MELAA	12 (2%)	10 (4%)
Asian	78 (16%)	46 (18%)
Unknown	3 (1%)	0 (0%)
Reason for referral		*p* < 0.01
PCB	231 (46.8%)	118 (46.8%)
Abnormal appearing cervix (no symptoms)	229 (46.4%)	110 (43.7%)
Abnormal appearing cervix and PCB	7 (1.4%)	16 (6.3%)
Abnormal bleeding (IMB & PMB)	24 (5.8%)	8 (3.2%)
Unable to take cytology	3 (0.6%)	0
Prior HPV test and/or cytology within 12 months of colposcopy appointment		*p* < 0.01
No HPV test	**483 (97.8%)**	**21 (8%)**
Normal cytology	467	13
Low grade cytology	13	1
No cytology	3	7
HPV other	**2 (0.4%)**	**40 (16%)**
Normal cytology	2	24
Low grade cytology	0	16
HPV not detected	**9 (1.8%)**	**191 (76%)**
Normal cytology	0	163
Low grade cytology	9	18
No cytology	0	10

Table 2 shows a comparative analysis between both cohorts. There was an increase in colposcopy FSAs following the introduction of an HPV‐based cervical screening programme. However, the absolute number and proportion of FSAs for clinical reasons reduced from 29% to 9% (*p* < 0.01). This reduction coincided with the increase in pre‐referral HPV testing (2% to 92%, *p* < 0.01) and the absence of high‐grade or malignant findings in the post‐HPV cohort.

**TABLE 2:** Comparative analysis between both cohorts.

	Pre‐HPV testing (2021–2023)	Post HPV‐testing (2023–2025)
Total number of FSAs	1699	2809
Total referrals for clinical reasons	494	252
% of All FSAs	29%	9%, *p* < 0.01
High‐grade/Malignant detected	7	0
Detection rate	1.4%	0%

All cases with high‐grade or malignant histology occurred in Cohort 1, when 7 people were diagnosed with high‐grade or malignant pathology. There were no people found to have high‐grade or malignant pathology detected in Cohort 2.

Table 3 summarises the histological outcomes of people referred to colposcopy. Most people referred for colposcopy for ‘clinical reasons only’, in both cohorts, did not have high‐grade or malignant disease. Biopsy rates were higher in Cohort 1 than in Cohort 2 (79% vs. 33%, *p* < 0.01). In Cohort 2 no PCB cases resulted in high‐grade lesions, whereas in Cohort 1 PCB accounted for most HSIL findings. Benign findings were categorised as either polyp or normal, with normal variants including ectropion, squamous metaplasia, nabothian cysts, scar tissue and inflammation. Among those assessed as having normal benign findings, biopsies were taken to support the colposcopic impression in 129/392 (33%) of individuals in Cohort 1 and 56/224 (25%) in Cohort 2. The biopsy rate was significantly lower in Cohort 2 (*p* = 0.049).

**TABLE 3:** Histological outcomes of people referred to colposcopy clinic for clinical reasons.

Cohort 1 (*n* = 494)				Cohort 2 (*n* = 252)			
Outcome by referral reason		Biopsy taken	No biopsy taken	Outcome by referral reason		Biopsy taken	No biopsy taken
PCB (*n* = 231)	Benign	74	135	PCB (*n* = 118)	Benign	25	88
	*Normal* ^a^	65	135		*Normal*	23	88
	*Polyp*	9	—		*Polyp*	2	—
	LSIL	18	—		LSIL	5	—
	HSIL	4	—		HSIL	—	—
	Cancer	—	—		Cancer	—	—
Abnormal bleeding (including PCB, PMB, IMB) and abnormal appearing cervix (*n* = 9)	Benign	6	1	Abnormal bleeding (including PCB, PMB, IMB) and abnormal appearing cervix (*n* = 16)	Benign	10	5
	*Normal*	3	1		*Normal*	7	5
	*Polyp*	3	—		*Polyp*	3	—
	LSIL	—	—		LSIL	1	—
	HSIL	—	—		HSIL	—	—
	Cancer	2	—		Cancer	—	—
Abnormal bleeding, excluding PCB (*n* = 24)	Benign	10	12	Abnormal bleeding, excluding PCB (*n* = 8)	Benign	2	5
	*Normal*	8	12		*Normal*	2	5
	*Polyp*	2	—		*Polyp*	—	—
	LSIL	2	—		LSIL	1	—
	HSIL	—	—		HSIL	—	—
	Cancer	—	—		Cancer	—	—
Abnormal appearing cervix only (*n* = 230)	Benign	105	115	Abnormal appearing cervix only (*n* = 110)	Benign	38	70
	*Normal*	53	115		*Normal*	24	70
	*Polyp*	52	—		*Polyp*	14	—
	LSIL	9	—		LSIL	2	—
	HSIL	1	—		HSIL	—	—
	Cancer	0	—		Cancer	—	—

^a^ Normal includes ectropion, inflammation, nabothian cyst, squamous metaplasia, atrophy, scar tissue, contact bleeding.

Table 4 describes the characteristics and outcomes of the 7 people diagnosed with high‐grade or malignant pathology in Cohort 1. Four were referred for PCB, one for an abnormal‐appearing cervix, one for an abnormal‐appearing cervix and PMB, and one for PCB and an abnormal‐appearing cervix. Two were diagnosed with invasive cervical cancer, 4 had cervical HSIL and 1 had VIN3. All 7 had had cytology performed prior to colposcopy referral; 6 had normal cytology and one had low‐grade cytology. Only one individual underwent pre‐colposcopy HPV testing, as they were being managed under the test‐of‐cure pathway after a previous high‐grade lesion.

**TABLE 4:** Characteristics and outcomes of the seven patients with high grade or malignant pathology.

Referral indication	Cytology prior to referral	HPV prior to referral	Final diagnosis	HPV status (at colposcopy)
Abnormal cervix and PCB	Normal	Not tested	Endocervical adenocarcinoma (cervix)	Not tested
Abnormal cervix + PMB	Normal	Not tested	Squamous cell carcinoma (cervix)	Not tested. (histological HPV‐associated SCC)
Abnormal cervix	Normal	Not detected	VIN3	Not tested
PCB	Low‐grade	Not tested	CIN3	Not tested
PCB	Normal	Not tested	CIN3	Not tested
PCB	Normal	Not tested	CIN3	Not tested
PCB	Normal	Not tested	CIN2	HPV16

## 4 Discussion

This study describes the pattern and outcomes of colposcopy referrals for clinical reasons at one tertiary hospital in AoNZ before and after the introduction of an HPV‐based cervical screening programme with the option of self‐administered tests. Referrals for clinical reasons decreased in absolute numbers and as a proportion of all referrals following the introduction of HPV testing. Following the introduction of HPV primary testing, there were no high‐grade or malignant lesions detected in those referred for colposcopy for clinical reasons and who didn't meet the screening guideline recommendations for colposcopy, compared to a 1.4% detection rate of high‐grade lesions prior to the introduction of testing. This suggests that HPV primary screening has reduced colposcopy clinic referral load for clinical indications, particularly among HPV‐negative women.

AoNZ was the first country to implement self‐administered HPV testing as the primary method of cervical screening, so this study assessing colposcopic outcomes of those referred for clinical reasons in this environment provides unique data [4]. Furthermore, a key strength of this study is the completeness of data within a large tertiary centre during early programme implementation, allowing detailed evaluation of real‐world outcomes.

Several interconnected factors likely contribute to the reduction in colposcopy referrals made for clinical reasons in patients who did not meet the screening test criteria for referral. Fewer people are having speculum examinations during cervical screening due to the shift to self‐administered HPV testing, meaning that incidental findings‐ such as a clinician perceived abnormal cervix or asymptomatic benign changes such as cervical polyp‐ are observed less frequently [9]. Clinicians may feel more reassured by an HPV not detected result and normal cytology, reducing the perceived need for immediate colposcopic assessment [10]. While we did not identify any high‐grade or malignant pathology among those referred to colposcopy for clinical reasons, because outcomes for symptomatic individuals who were not referred were not captured, we cannot be certain that all significant pathology was identified.

Local service pressures, including limited availability of general gynaecology appointments and regional variation in access, may influence both the setting in which symptomatic individuals are assessed (general gynaecology vs. colposcopy clinics) and clinicians' decisions to refer to colposcopy or manage in primary care [11]. In some centres, presentations such as cervical polyps or PCB are routinely managed within general gynaecology services, whereas in regions with prolonged gynaecology waitlists, clinicians may preferentially refer individuals with abnormal‐appearing cervixes to colposcopy as a more reliable route to specialist gynaecologist review [12]. These variations highlight the importance of nationally consistent referral pathways and clear education regarding features of a concerning cervical appearance [12, 13]. The reduction in referrals for clinical reasons occurred in the context of a significant increase in colposcopy FSAs following the introduction of HPV screening. This is consistent with international HPV‐screening transitions, where a more sensitive and acceptable test initially uncovers prevalent disease before referral rates stabilise [14].

It is reassuring that no individual in Cohort 2 who was referred solely for clinical indications was found to have high‐grade or malignant disease, although the small number of events limits the study's power to detect a statistically significant difference in this uncommon outcome. The clinical implications of these patterns are important. In Cohort 2, 76% of people referred for clinical indications alone had HPV not detected results, and none had high‐grade or malignant pathology found. This is reassuring and supported by long‐term evidence showing that the risk of developing CIN2+ after a negative HPV test is less than 1% over 5–10 years [15]. In contrast, all high‐grade or malignant lesions in our study occurred in the pre‐HPV cohort despite most having normal cytology, and almost all were HPV‐related conditions, including two invasive cancers. Although HPV testing was not performed on the 4 individuals who underwent LLETZ, their CIN2+ diagnoses are strongly associated with high‐risk HPV, suggesting that these lesions would have likely been identified through HPV‐based primary screening. The single case of VIN3 was an incidental finding unrelated to the referral indication or cervical pathology. This person had previously tested positive for HPV and their VIN3 was of the usual, HPV‐associated type. As cervical screening is not intended to detect vulval disease, its identification in this study should not be interpreted as a missed screening opportunity or a limitation of HPV primary testing [16].

While these findings are reassuring, clinical vigilance remains essential. Symptomatic women or those with an abnormal‐appearing cervix require careful evaluation, irrespective of the co‐test result. HPV testing will not detect rare HPV‐independent cervical cancers, such as gastric‐type adenocarcinoma and metastatic non‐HPV related disease [17]. As extended vaccination takes effect and we move closer to elimination, the proportion of cervical malignancies that are not HPV related will likely increase. Clinicians must therefore balance reassurance from HPV not detected results with ongoing clinical judgement, ensuring that people with persistent or recurrent symptoms, or concerning examination findings, are not inadvertently under‐investigated [17].

A limitation is that this study was conducted at a single tertiary centre with a relatively short post‐implementation follow‐up period, and findings may not be fully generalisable across regions or time. Symptoms such as PCB and findings such as cervical polyps are managed outside of colposcopy clinics in some centres. People with symptoms who were managed outside colposcopy were not captured, and longer‐term surveillance will be necessary to confirm that the lack of high‐grade or malignant pathology at colposcopy reflects the absence of disease in this cohort rather than disease that was not detected or subsequently diagnosed. Outcome status was assessed only at the time of discharge from colposcopy. Therefore, it is unknown whether any individuals later developed cervical cancer or required further intervention elsewhere in the health system. Future multi‐centre research should examine longitudinal outcomes of symptomatic HPV‐not detected people managed conservatively, evaluate patient experience, and incorporate cost‐effectiveness analyses to inform national triage policy.

In Cohort 2, 8% had not had HPV testing prior to referral. This group likely represents individuals who were referred prior to HPV becoming the primary screening test and were still awaiting their FSA at the time of Cohort 2 data collection.

Colposcopy is an invasive, anxiety‐provoking and resource‐intensive procedure; therefore, reducing unnecessary referrals spares people from avoidable interventions and allows specialist services to focus on higher‐value assessments [7]. The findings from this study suggest an opportunity to preserve colposcopy's capacity by refining clinical referral pathways, especially for symptomatic people with HPV not detected results and normal cytology. Establishing clear criteria for colposcopy versus general gynaecology referral would enhance both service efficiency and diagnostic safety [18].

Furthermore, as self‐testing reduces direct visualisation of the cervix, nationally consistent guidance for primary care on recognising concerning cervical appearances and appropriate referral thresholds will become increasingly important. Clear, symptom‐based triage and safety‐netting may reduce unnecessary referrals for benign findings while ensuring that persistent symptoms or concerning appearances are appropriately investigated. As referral patterns evolve, low‐risk, HPV‐not‐detected symptomatic individuals may increasingly be managed in primary care or general gynaecology, with clear pathways for escalation.

HPV primary screening has reduced referrals to colposcopy for clinical reasons, with no high‐grade or malignant pathology detected among those referred who did not meet screening‐based referral criteria. While these findings are reassuring, the outcomes of symptomatic individuals managed outside colposcopy were not captured, and longer‐term follow‐up is needed to confirm these findings. HPV‐negative results should therefore not replace clinical assessment, particularly for persistent or concerning symptoms or abnormal cervical findings, given that rare HPV‐independent cervical malignancies will not be detected by HPV testing [18, 19].

## Funding

This project was funded as a University of Otago Summer Studentship sponsored by ISTAR.

## Conflicts of Interest

The authors declare no conflicts of interest. All authors have reviewed and agreed to this statement.

## Data Availability

The data that support the findings of this study are available from the corresponding author upon reasonable request.
